# Supplementary material for: BDNF Spinal Overexpression after Spinal Cord Injury Partially Protects Soleus Neuromuscular Junction from Disintegration, Increasing VAChT and AChE Transcripts in Soleus but Not Tibialis Anterior Motoneurons
Source: Biomedicines. 2022 Nov 8;10(11):2851. doi: 10.3390/biomedicines10112851 (PMC9687248; doi:10.3390/biomedicines10112851)
Supplement: Supplementary file 1 [file biomedicines-10-02851-s001.zip › biomedicines-1924104-supplementary/Supplementary Figure S1 description.pdf]

Figure S1. Overexpression of recombinant BDNF in primary hippocampal cell cultures transduced with AAV1/2-BDNF leads to a production and release of biologically active protein.

A. Above: A topographic organization of rat BDNF PCR product. PCR reaction was performed with high-fidelity DNA polymerase (Phusion Polymerase, FINNZYMES); template was pExpress-1 carrying complete coding sequence (cds) of rat BDNF. The construct contained a *c-myc* tag to identify the transgenic protein in SCT-BDNF rats.
Below: A schematic map of AAV plasmid containing BDNF sequence introduced downstream human synapsin (hSyn, pAAV-hSyn-BDNF) promoter to target recombinant BDNF protein expression to neurons. Vector also contained the Woodchuck hepatitis post-transcriptional response element (WPRE) to augment transgene expression and bovine growth hormone polyadenylation signal (bGHpAITR is 145nt long inverted terminal repeat, the only one autonomic to AAV virus DNA fragment, responsible for DNA packaging into the viral capsid.

B. A representative Western blot demonstrating mature BDNF transgenic protein with *a c-myc* tag (~16 kDa; detected with anti-c-Myc epitope Ab), produced ([hsyn-BDNF] lanes 1, 2, upper panel), and released to the culture medium (lanes 1, 2, lower panel) by AAV-BDNF-transduced cells 6 days after transduction (DIV 12). Naive, control cells treated with the hsyn-BDNF conditioned medium (30 min) show a faint signal suggesting small uptake of the transgenic protein from the medium (lanes 3, 4). Non-transfected cells from BDNF-treated and non-treated cultures served as a negative control for transgenic BDNF detection (lanes 5, 6).

The levels of phosphorylated form of TrkB receptor (pTrkB) and of S6 ribosomal protein (BDNF-TrkB downstream target in a mTOR-dependent pathway), are increased in non-transfected control cells treated with the hsyn-BDNF conditioned medium (lanes 3, 4) as compared to the untreated cells (lane 6). Control cells treated with human recombinant BDNF protein (50 ng/mL) served as a positive control for TrkB stimulation (lane 5).

Western blot: Primary hippocampal cells were lysed in SDS lysis buffer (50mM Tris pH 8.0, 50 mM SDS, 1x Complete Protease Inhibitor Cocktail, Roche, 1% of NP40 and 1x PhosStop Phosphatase Inhibitor Cocktail (Roche)), centrifuged and supernatants (30ug protein) were separated by SDS-PAGE (10% polyacrylamide gels) and analysed with immunoblot. After protein transfer, PVDF membranes were blocked for 1h at RT with 5% bovine serum albumin, cut into stripes, each incubated with respective primary antibody diluted in blocking solution overnight. Primary antibodies (Ab) were then detected with horseradish peroxide (HRP) conjugated secondary antibodies with ECL reagent as a substrate. β-tubulin was used to verify equal loading of samples.

The following primary antibodies were used: c-Myc (#2272s Rb, Cell Signaling Technology, 1:500), Phospho-TrkB (pTyr705) (#11328, Rb, Signalway, 1:400), Phospho-S6 ribosomal protein (# 4858 Rb, Cell Signaling Technology,1:500), β-tubulin clone (#3873, Cell Signaling, 1:1000). Secondary Abs: Gt anti-Rabbit HRP (# P0448 DAKO, 1:10000), Gt anti-Mouse HRP (# P0447 DAKO, 1:10000). Protein Ladder Biotinylated + Anti-Biotin HRP Linked Ab (1:1000, Cell Signaling Kit #7727).
