# Supplementary material for: BDNF Spinal Overexpression after Spinal Cord Injury Partially Protects Soleus Neuromuscular Junction from Disintegration, Increasing VAChT and AChE Transcripts in Soleus but Not Tibialis Anterior Motoneurons
Source: Biomedicines. 2022 Nov 8;10(11):2851. doi: 10.3390/biomedicines10112851 (PMC9687248; doi:10.3390/biomedicines10112851)
Supplement: Supplementary file 1 [file biomedicines-10-02851-s001.zip › biomedicines-1924104-supplementary/Supplementary Figure S2 description.pdf]

Figure S2. Sequential screenshots of ImageJ TEM analysis.

The upper panel presents the analysis of the distance of the synaptic cleft and of the number of active zones.

1. TEM image of NMJ selected for the analysis.
2. Marking of a continuous presynaptic membrane fragment for manual measurement. For counting of the density of AZ, the selection of images was based on the criterion of the smallest visible length of the presynaptic membrane equal to 1µm/image.
3. Synaptic cleft distance was a manually measured inner width between presynaptic membrane and postsynaptic crests of junctional folds. The average value of five measurements was taken for further analysis. Eight to 24 images per group were analyzed.
4. The number of AZs (circles) was calculated manually.

The lower panel presents an analysis of synaptic vesicles.

The synaptic bouton area of 0.6-1 μm^2 was used for calculation of the SVs density parameter. SVs density was measured in the field of at least 500 nm^2 remaining after exclusion of the area occupied by the axonal mitochondria.
