# Supplementary material for: BDNF Spinal Overexpression after Spinal Cord Injury Partially Protects Soleus Neuromuscular Junction from Disintegration, Increasing VAChT and AChE Transcripts in Soleus but Not Tibialis Anterior Motoneurons
Source: Biomedicines. 2022 Nov 8;10(11):2851. doi: 10.3390/biomedicines10112851 (PMC9687248; doi:10.3390/biomedicines10112851)
Supplement: Supplementary file 1 [file biomedicines-10-02851-s001.zip › biomedicines-1924104-supplementary/Supplementary Figure S3A description.pdf]

Figure S3A. Representative confocal images of endogenous and transgenic BDNF protein expression in the lumbar spinal cord of SCT-BDNF rats 2 weeks after spinal cord transection and AAV-BDNF injection.

The images show maximal intensity projections from 11-20 optical slices. Transgenic protein (c-Myc IF) is detected in neuronal perikarya and fibers in L1 segment, in the proximity of AAV-BDNF injection site, and in neuronal fibers in L2 and in L4-5, at the Sol and TA MN vicinity. No c-Myc IF signal is detected in tissue from Control and SCT-PBS rats.

BDNF transgenic protein with c-Myc tag was detected with murine anti c-Myc antibody (1:200, no. 2276, Cell Signaling), BDNF protein was detected with rabbit antibody (1:200, ANT-010, Alomone Labs). The images of MNs were captured with Zeiss LSM800 Airyscan confocal microscope (Carl Zeiss, Jena, Germany) using PL APO 63x/1.4 Oil DIC objective. The Z stacks of the 16 bit images consist of 11-20 digital slices collected with Airyscan Detector (32x GaAsP detectors) at 0.5 μm intervals with a pixel size of 0.04 μm and scan resolution of 4096 x 4096 pixels. Images were collected at constant exposure parameters for each of three channels detecting fluorescence labeling for BDNF, c-Myc, and tracer with the use of the diode lasers (405nm, 488nm, 561nm, 640nm).
