# Supplementary material for: BDNF Spinal Overexpression after Spinal Cord Injury Partially Protects Soleus Neuromuscular Junction from Disintegration, Increasing VAChT and AChE Transcripts in Soleus but Not Tibialis Anterior Motoneurons
Source: Biomedicines. 2022 Nov 8;10(11):2851. doi: 10.3390/biomedicines10112851 (PMC9687248; doi:10.3390/biomedicines10112851)
Supplement: Supplementary file 1 [file biomedicines-10-02851-s001.zip › biomedicines-1924104-supplementary/Supplementary Figure S3B description.pdf]

Figure S3B. Representative confocal images of TrkB and p75 protein expression in the lumbar spinal cord in the Control, SCT-PBS and SCT-BDNF rats 2 weeks after spinal cord transection and AAV-BDNF injection.

Upper panel: TrkB immunostaining detected TrkB-positive neuronal fibers (exemplified by empty white arrows), punctate signal (exemplified by solid white arrows) and neurons with cytoplasmic signal. Panel marked with asterisk shows brighter neurons that appeared in other spinal cord slices from SCT-PBS group. **Bottom panel:** Images show the pattern and intensity of p75 immunostaining of large diameter neurons and surrounding neuropil in the ventral horn of the spinal cord. Note a remarkable increase of p75 labeling intensity in SCT-PBS group, and weaker signal in SCT-BDNF group.

The images show maximal intensity projections from 10-15 optical slices. TrkB receptor was detected with Rb antibody (ANT-019, Alomone Labs, 1:200), p75 protein was detected with Rb antibody (ANT-007, Alomone Labs, 1:500). The images were captured with Zeiss LSM 780 confocal microscope (Carl Zeiss, Jena, Germany) using PL APO 40x (1.4 NA) DIC oil-immersion objective. The Z stacks of the 16 bit images consist of 10-15 digital slices collected at 0.21 μm intervals with a pixel size of 0.069 μm. Images were collected at constant exposure parameters for each marker with the use of the 561 nm diode-pumped solid-state laser, the 633 nm helium neon laser, and the 488 nm argon laser.
