# Supplementary material for: BDNF Spinal Overexpression after Spinal Cord Injury Partially Protects Soleus Neuromuscular Junction from Disintegration, Increasing VAChT and AChE Transcripts in Soleus but Not Tibialis Anterior Motoneurons
Source: Biomedicines. 2022 Nov 8;10(11):2851. doi: 10.3390/biomedicines10112851 (PMC9687248; doi:10.3390/biomedicines10112851)
Supplement: Supplementary file 1 [file biomedicines-10-02851-s001.zip › biomedicines-1924104-supplementary/Supplementary Figure S4 description.pdf]

Figure S4. The effect of AAV-BDNF injection to the lumbar L1-2 spinal segment on (A) NT-3 mRNA and (B) TrkC mRNA level in the L3-6 spinal segments and muscles 2 weeks after spinal cord transection. *nt-3* and *trkC* mRNA levels were measured with a qPCR assay. Box and whisker plots show the minimum and maximum score (whiskers), first and third quartile (box), median (line), and mean (x). Data are from 5 Control, 6 SCT-PBS and 5 SCT-BDNF rats (L3-6 segments); 9-10 Control, 4-6 SCT-PBS and 5 SCT-BDNF rats (muscles). The data of *nt-3* mRNA measurements present results obtained with a use of a UPL TaqMan probe (#73) which makes no distinction between 4 transcript variants, where 4th variant constitutes 98% of total amount of transcripts (for detailed characteristics see [1]). The use of #29 probe recognizing 1-3 variants showed no difference in their level between groups (not shown). The *Mann-Whitney U* test was used (*p≤0.05) to assess differences between experimental groups.

1. Gajewska-Woźniak, O.; Skup, M.; Kasicki, S.; Ziemlińska, E.; Czarkowska-Bauch, J. Enhancing proprioceptive input to motoneurons differentially affects expression of neurotrophin 3 and brain-derived neurotrophic factor in rat hoffmann-reflex circuitry. *PLoS One* **2013**, *8*, e65937, doi:10.1371/journal.pone.0065937.
