# Supplementary material for: BDNF Spinal Overexpression after Spinal Cord Injury Partially Protects Soleus Neuromuscular Junction from Disintegration, Increasing VAChT and AChE Transcripts in Soleus but Not Tibialis Anterior Motoneurons
Source: Biomedicines. 2022 Nov 8;10(11):2851. doi: 10.3390/biomedicines10112851 (PMC9687248; doi:10.3390/biomedicines10112851)
Supplement: Supplementary file 1 [file biomedicines-10-02851-s001.zip › biomedicines-1924104-supplementary/Supplementary Figure S5 description.pdf]

Figure S5. Representative confocal images of Periaxin, Neurofilament and nAChR protein expression in NMJs of TA and Sol in the Control, SCT-PBS and SCT-BDNF rats 2 weeks after spinal cord transection and AAV-BDNF injection.

In animals subjected to spinal cord transection, periaxin staining did not cover terminal nerve branches and appeared to be more dispersed than in intact rats. This observation was more pronounced in NMJs of SCT-PBS rats than in SCT-BDNF rats. Single Periaxin channel view from the site where insulated nerve starts to branch into the NMJ is shown in the upper right panel for each image.

The images show maximal intensity projections from 7-21 optical slices. Periaxin was detected with Rb antibody (Peter Brophy, Centre for Neuroscience Research, University of Edinburgh, UK, 1:3000), Neurofilament protein was detected with Ms monoclonal antibody (M0762, DAKO, 1:200). The images were captured with Zeiss LSM 780 confocal microscope (Carl Zeiss, Jena, Germany) using PL APO 40x (1.4 NA) DIC oil-immersion objective. The Z stacks of the 16 bit images consist of 7-40 digital slices collected at 0.21 μm intervals with a pixel size of 0.069 μm. Images were collected at constant exposure parameters for each marker with the use of the 561 nm diode-pumped solid-state laser, the 633 nm helium neon laser, and the 488 nm argon laser.
