# Supplementary material for: BDNF Spinal Overexpression after Spinal Cord Injury Partially Protects Soleus Neuromuscular Junction from Disintegration, Increasing VAChT and AChE Transcripts in Soleus but Not Tibialis Anterior Motoneurons
Source: Biomedicines. 2022 Nov 8;10(11):2851. doi: 10.3390/biomedicines10112851 (PMC9687248; doi:10.3390/biomedicines10112851)
Supplement: Supplementary file 1 [file biomedicines-10-02851-s001.zip › biomedicines-1924104-supplementary/Supplementary Table S1.pdf]

Table S1. List of probes and primers

Gene symbol	Name of coded protein	Accession number	Roche UPL No. and sequence	Forward primer (5'–3')	Reverse Primer (5'–3')	Amplicon length/nt
Ache	AchE	NM_172009.1	25 tggaggag	cctggatccctcactgaact	tgcataagtcgctgagcaaa	60
Bdnf	BDNF	NM_012513.4	67 tgctggag	gcagtcaagtgcctttggag	cggcatccaggtaatttttg	69
Chat	ChAT	NM_001170593.1	106 ctctggct	aaggtcgggtggacaacatc	cagcagctgcagtttctcag	110
Ntrk2	TrkB	NM_012731.3<br>NM_001163168.3	80 cctggaga	cgccaccttgacttgtctg	agtgtcggggctggattt	108
Ntrk2	TrkB FL	NM_012731.3	106 ctctggct	tgggaaatggaaaccagaa	tgggttttcctatgcaggac	110
Slc18a3	VAChT	NM_031663.2	64 ccaggctg	cactctcttggctttgagca	actggtgcgtagagcaggtt	65
Chrm2	M2	AB017655.1	64 ccaggctg	cactctcttggctttgagca	actggtgcgtagagcaggtt	65
Chrm4	M4	NM_031547.1	56 tgctgtcc	cctggtgactgttgtgggta	tgcaactgcctgttgacct	63
Mbp v1-5	MBP	NM_001025291.1<br>NM_001025292.1<br>NM_001025293.1<br>NM_001025294<br>NM_017026.2	56 tactatcc	ggactgcaggagttctctgg	gctgaccaccctaaagtgaga	68
S100b	S100	NM_013191.1	65 ctggagga	gaaggagctcatcaacaacga	tccatcactttgtccaccac	78
