# Supplementary figures and images for: BDNF Spinal Overexpression after Spinal Cord Injury Partially Protects Soleus Neuromuscular Junction from Disintegration, Increasing VAChT and AChE Transcripts in Soleus but Not Tibialis Anterior Motoneurons
Source: Biomedicines. 2022 Nov 8;10(11):2851. doi: 10.3390/biomedicines10112851 (PMC9687248; doi:10.3390/biomedicines10112851)

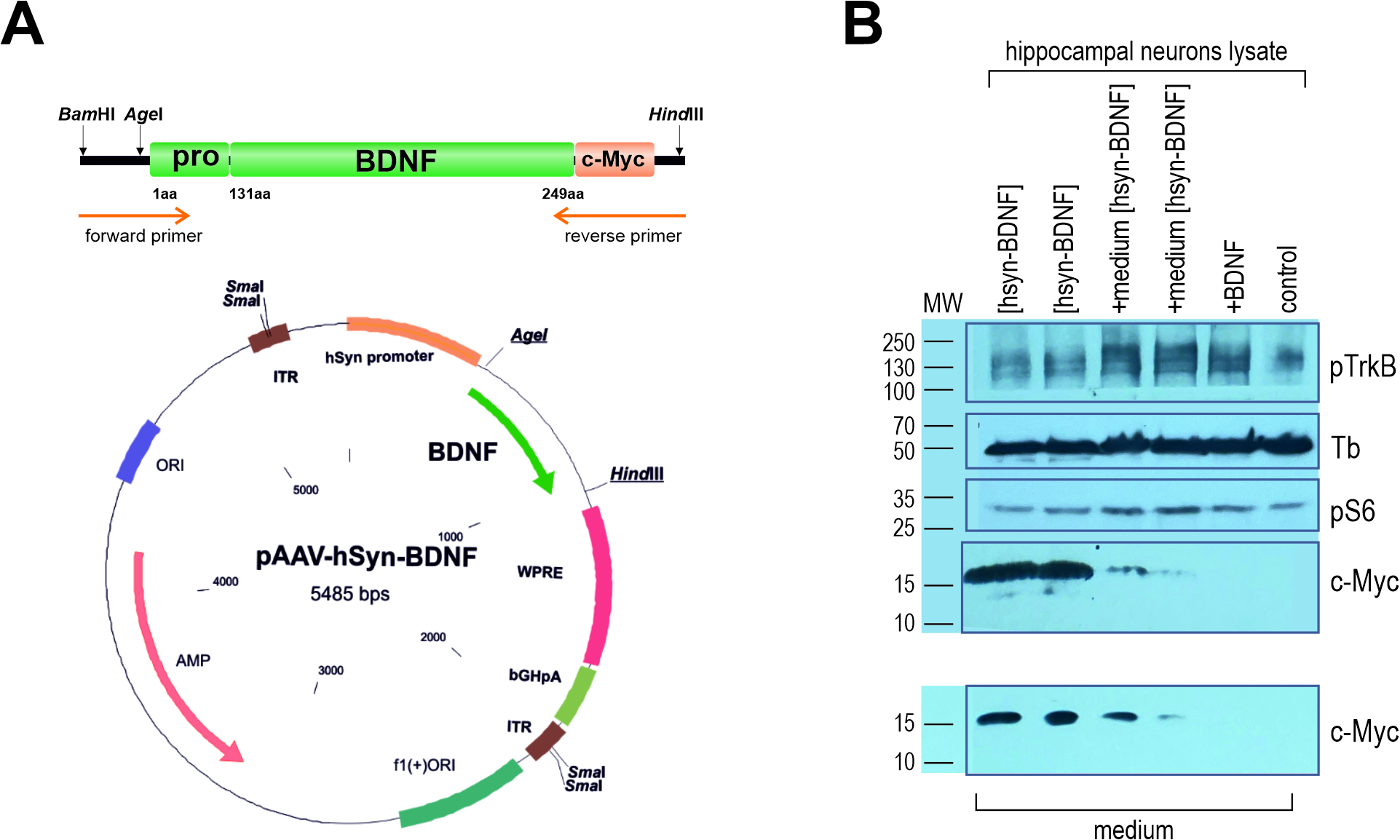

Supplement: Supplementary file 1 [file biomedicines-10-02851-s001.zip › biomedicines-1924104-supplementary/Supplementary Figure S1.tif]

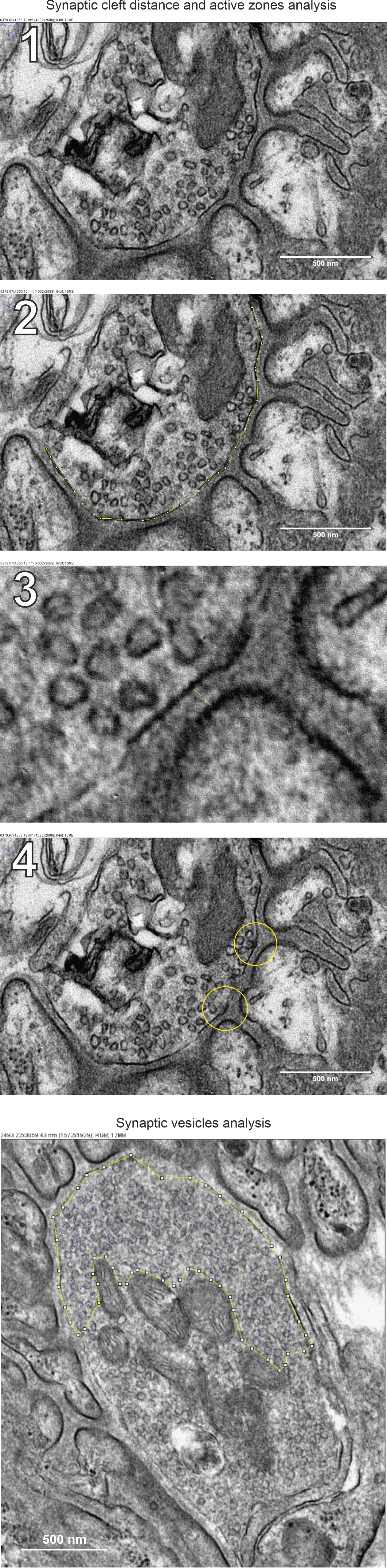

Supplement: Supplementary file 1 [file biomedicines-10-02851-s001.zip › biomedicines-1924104-supplementary/Supplementary Figure S2.png]

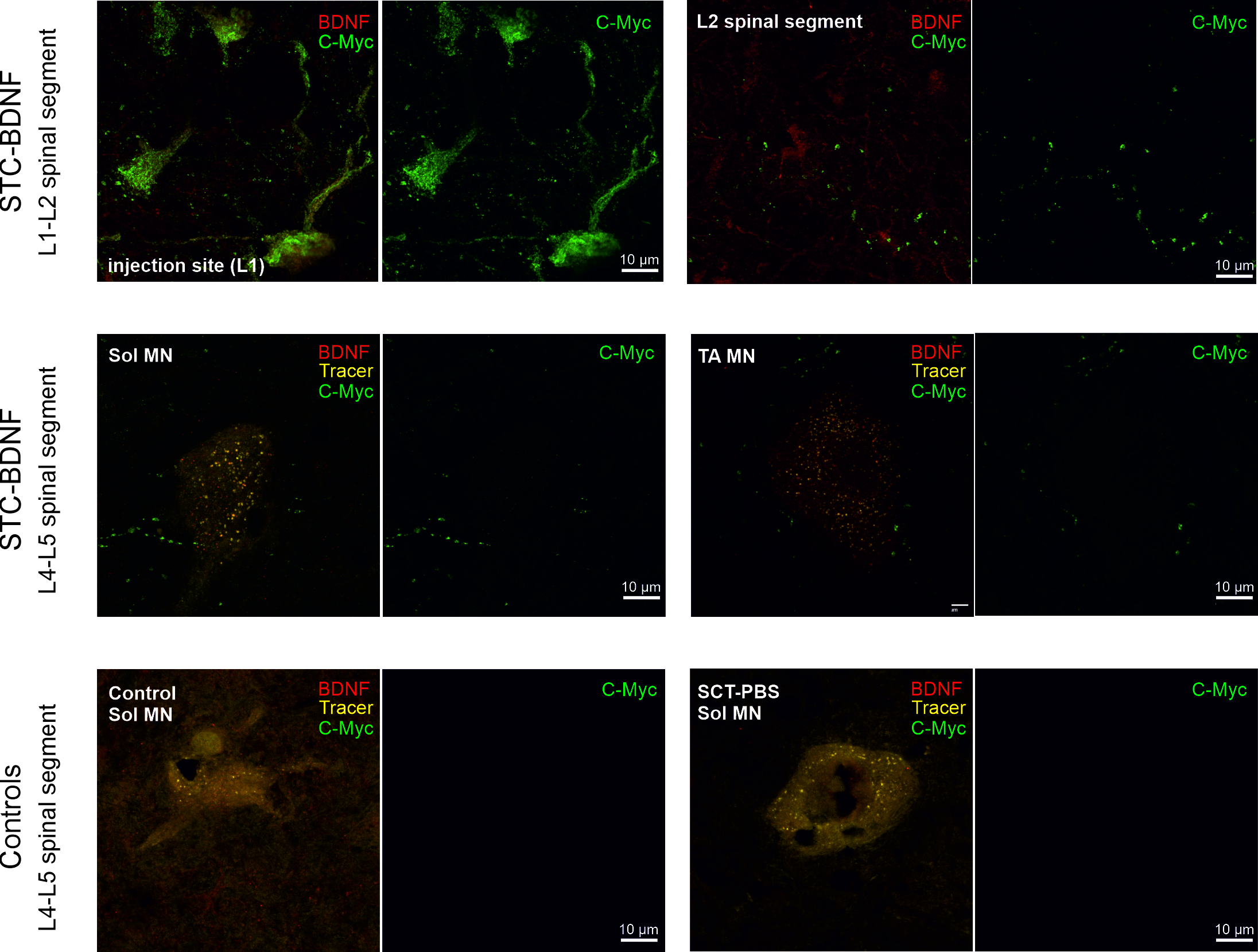

Supplement: Supplementary file 1 [file biomedicines-10-02851-s001.zip › biomedicines-1924104-supplementary/Supplementary Figure S3A.tif]

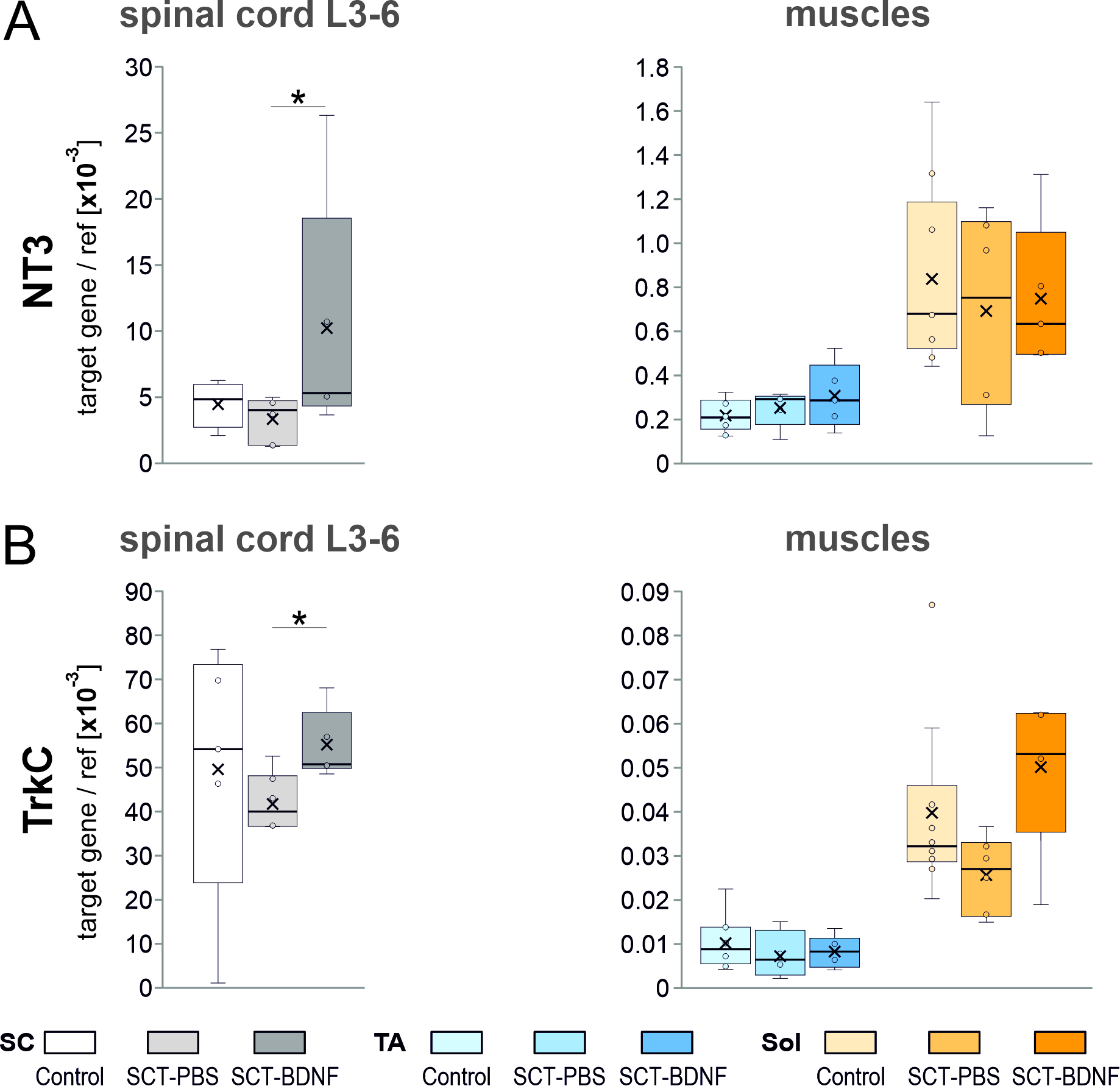

Supplement: Supplementary file 1 [file biomedicines-10-02851-s001.zip › biomedicines-1924104-supplementary/Supplementary Figure S4.tif]

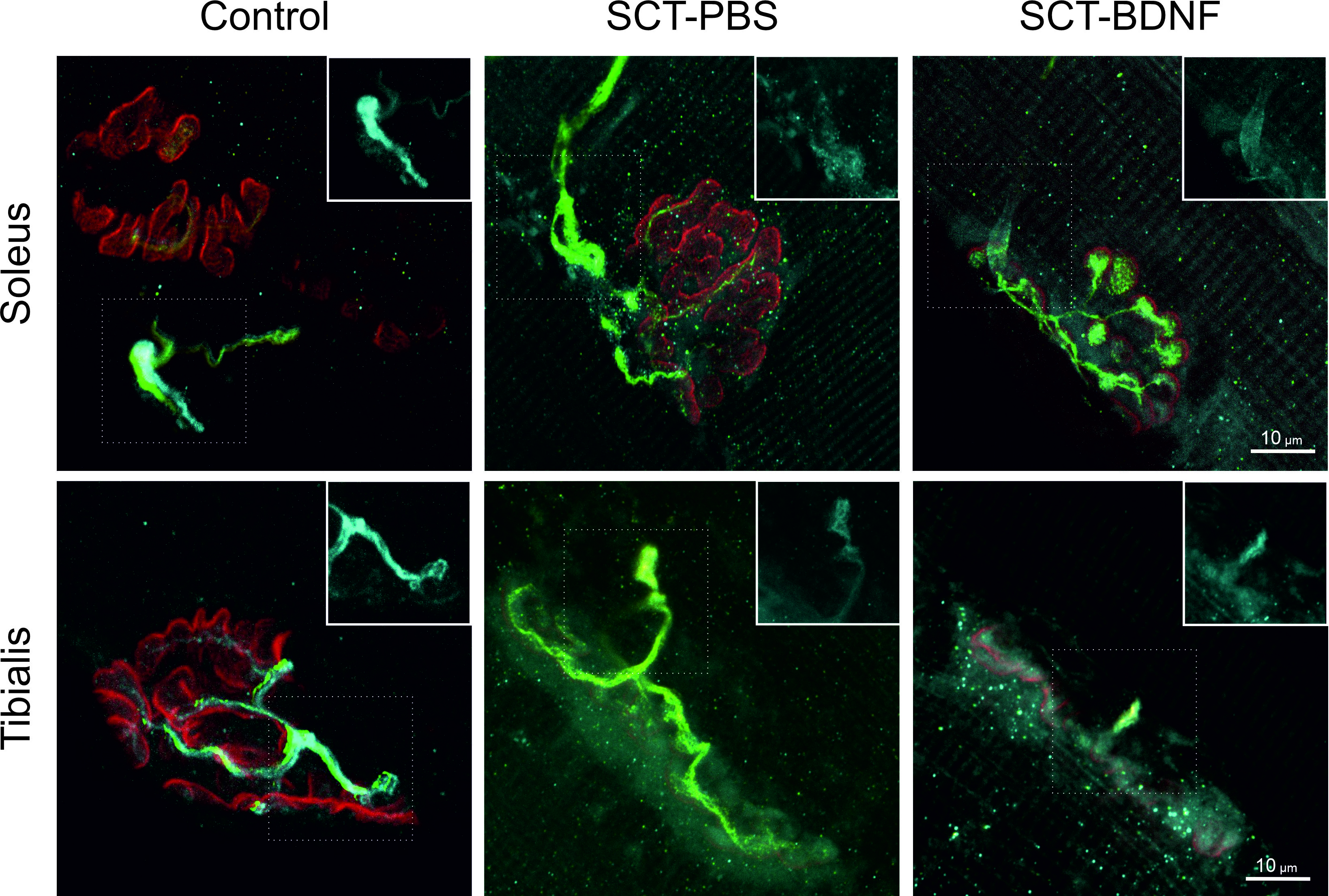

Supplement: Supplementary file 1 [file biomedicines-10-02851-s001.zip › biomedicines-1924104-supplementary/Supplementary Figure S5.jpg]
